# Cerebellar Cavernous Malformation (Cavernoma): A Case Report

**DOI:** 10.7759/cureus.4371

**Published:** 2019-04-03

**Authors:** Oscar Cisneros, Razia Rehmani, Katherine Garcia de de Jesus

**Affiliations:** 1 Internal Medicine, St. Barnabas Hospital Health System / Albert Einstein College of Medicine, Bronx, USA; 2 Neuroradiology, St. Barnabas Hospital Health System, Bronx, USA

**Keywords:** cavernoma, congenital, vascular malformation, deep venous anomaly

## Abstract

Cavernous malformations are congenital or acquired vascular abnormalities. They are uncommon entities with an incidence of 0.5% of the general population and usually are unnoticed until a hemorrhagic event occurs. Cavernomas can be concurrently seen with developmental venous anomalies (DVAs) in 20% (range 20%-40%) of cases, in which case they are known as mixed vascular malformations. We report a case of a healthy young adult, who presented with acute onset of headache, dizziness, and nausea with intermittent episodes of vomiting for four days. Brain tomography imaging at presentation revealed likely multiple foci of intracranial hemorrhage; however, magnetic resonance imaging (MRI) showed findings suggestive of an underlying cavernoma that had bled, in addition to a coexisting DVA. The patient was discharged home with no deficits. Outpatient follow-up five months later revealed no symptoms or neurologic deficits.

## Introduction

Cavernous malformations are a rare type of cerebral vascular malformation. These are slow-flow vascular structures that undergo recurrent hemorrhage and are characterized by low and high intensities on magnetic resonance imaging (MRI) [1,2]. With the advances of non-invasive imaging technology, these types of malformations are more frequently detected. Intracranial developmental venous anomalies (DVAs) are congenital abnormalities of venous drainage in which blood flows at slow velocity. DVAs can occur sporadically as well [3]. Herein we describe a case of a cavernoma in a young patient presenting with headache and dizziness.

## Case presentation

**Figure 1:**
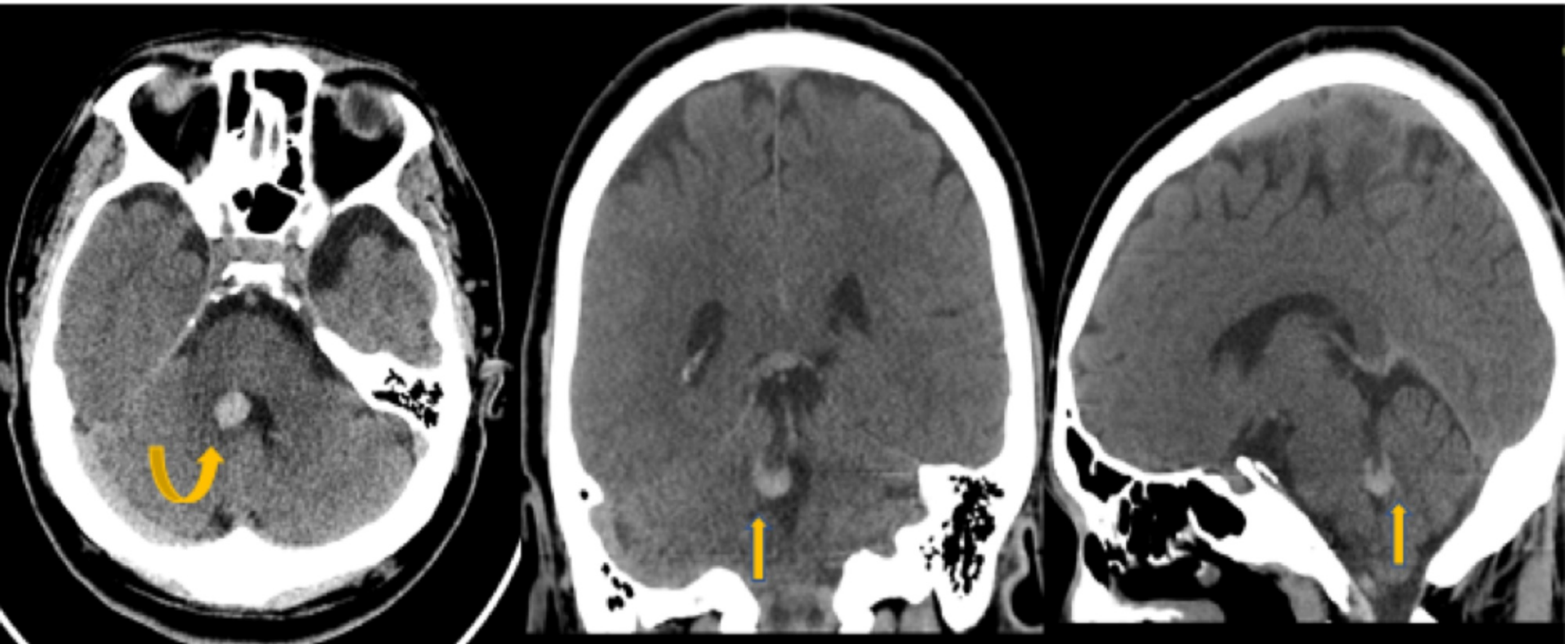
Non-contrast Head Computed Tomography (CT) Non-contrast head CT images demonstrate a focal area of hyperdensity about the medial aspect of the right cerebellum adjacent to the fourth ventricle (yellow arrows).

**Figure 2:**
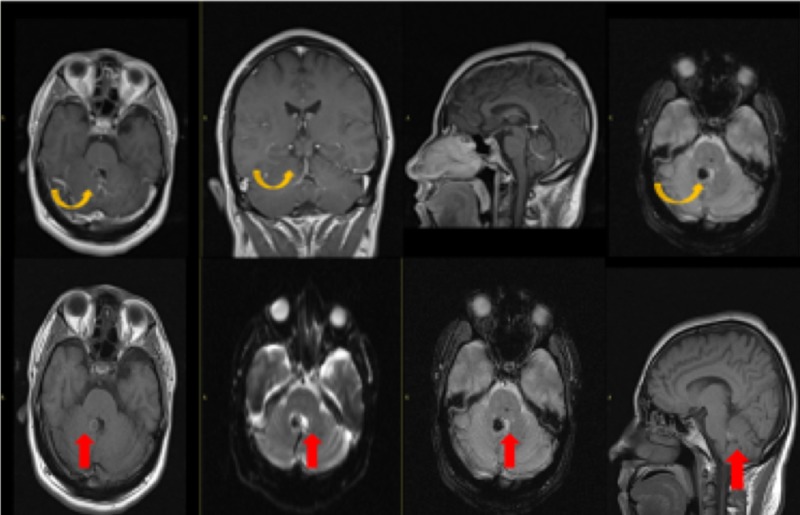
Magnetic Resonance Imaging with Contrast Post-contrast images demonstrate a focal area of blooming at the previously seen site of high density on CT at the right medial margin of the fourth ventricle without enhancement on post-contrast (yellow arrow) representing cavernoma. There is also demonstration of caput medusae appearance of small branching veins draining into a single vein adjacent to this lesion (red arrow) suggestive of a developmental venous anomaly (DVA).

We present a 35-year-old Hispanic male with no significant medical history and a social history remarkable for occasional cocaine and marihuana use who presented complaining of a right-sided parietal headache and dizziness. A review of his systems was also positive for intermittent nausea and vomiting for four days. Examination revealed an alert and oriented patient, with no focal deficits appreciated, moving all extremities with sensation grossly intact. Lung auscultation and abdominal examination did not disclose any abnormal findings. His laboratory workup was unremarkable, without significant electrolyte imbalances noted. A computed tomography (CT) scan of the brain was done, which revealed intracranial hemorrhages, with a prominent 12 mm hemorrhagic component layering along the right side of the fourth ventricle, trace hemorrhage along the bilateral tentorium and posterior interhemispheric fissure as well as a punctate hemorrhagic focus about the left paracentral frontal lobe, as can be seen in Figure 1. There was also demonstration of caput medusae appearance of small branching veins draining into a single vein adjacent to the lesion, suggestive of a deep venous anomaly. Brain MRI revealed an isolated rounded lesion in the right cerebellum adjacent to the fourth ventricle measuring up to 10 mm suggestive of a cavernous hemangioma as the primary cause of bleeding, as can be seen in Figure 2. The patient’s symptoms resolved over a period of two months following onset and he continued without development of neurologic symptoms. The treating physicians elected to continue to monitor the patient clinically and surgical resection was deferred.

## Discussion

Cavernous malformations are congenital or acquired vascular anomalies that occur in approximately 0.5% of the general population. Patients are usually asymptomatic but may present after an acute or recurrent hemorrhage usually around 40 to 60 years of age. Initial presenting features may include seizures, headaches, and neurologic deficits [1,2,4,5,6]. Our patient likely had dizziness due to localization of the bleeding of the cavernoma in the right side of the cerebellum.

MRI is considered the most sensitive and specific modality for detecting cavernomas [3]. It has been described that up to 55% of patients will have an affected relative. A study in Hispanic-American kindred showed strong evidence linking cavernous malformations to a segment of 7q in all families [7]. Regamonti et al. also described familial occurrence [8]. Genetic studies were not done in our patient; however, it would likely be indicated due to high incidence described in some populations.

Cavernomas tend to be supratentorial (80%) and are usually solitary. One third of patients with sporadic lesions may have more than one [3]. Larger lesions appear as foci of hyperdensity on CT. Smaller lesions, however, may not be seen on initial CT, unless there has been a recent bleed, in which case edema may be seen around the lesion.

On MRI, cavernomas are better evidenced using special techniques such as gradient echo (GRE) or susceptibility weighted imaging (SWI). They usually have a characteristic popcorn, ball-like appearance with a low-signal-intensity rim due to hemosiderin deposition [2-4,9]. Subacute hemorrhage and degraded blood products within the lesion produce a halo of signal hyperintensity around the lesion on T-1 weighted images. This in particular is useful in differentiating cavernomas from other intracranial hemorrhages [4]. These lesions do not enhance, although enhancement is possible and are angiographically occult.

Cavernomas can be concurrently seen with DVAs in 20% of cases (range 2%-40%). When this occurs, they are known as mixed vascular malformations [1]. In our patient, brain imaging revealed numerous abnormal blood vessels noted to be radiating from the cerebellar lesion, a finding that is highly suggestive of DVAs associated with cavernoma. However, further diagnostic imaging such as cerebral angiograms are needed to confirm this coexistence. DVAs are now considered to be the most common cerebral vascular malformation accounting for 55% of all cases in this era of post-contrast cross-sectional imaging. They are a slow-flow venous anomaly consisting of numerous dilated medullary veins converging into a single vein forming the classic caput medusae or palm tree appearance, which in turn drains into a dural sinus or ependymal vein. DVAs are usually incidental findings but may present with intracranial hemorrhage with vague neurologic symptoms such as headache or dizziness. The cause of cerebral hemorrhage in patients with DVAs is usually attributed to the cavernoma.

Dystrophic calcifications may be seen in up to 30% of cases. DVAs demonstrate the classic appearance on post-contrast MRI and CT. Susceptibility weighted imaging is the preferred sequence for low flow venous anomalies. Angiography also demonstrates the classic caput medusae appearance during the venous phase with normal arterial phase and no shunting. No differential diagnosis is usually offered since DVAs tend to have a classic appearance. ﻿Cerebral DVAs can also be associated with head and neck venous malformations [10].

Isolated DVAs require no treatment. Asymptomatic cavernomas can be treated conservatively. Symptomatic cases presenting with hemorrhage, edema with resultant mass effect and epileptic activity may require surgery. In these symptomatic cases, when possible, complete resection is curative. The current established indications for surgical management are overt hemorrhage, focal neurologic symptoms, and/or medically intractable epilepsy. It is imperative to inform the surgeon of the presence of associated DVA, since cauterization of collecting vein can result in venous infarct of the area of brain being drained.

## Conclusions

This case presents a patient with intracranial hemorrhage with an underlying cavernoma and likely associated DVAs. Cavernous malformation is an uncommon entity that might coexist with deep venous anomalies, therefore making management more challenging. The brain imaging findings and clinical presentation in our patient suggest that the cavernoma represents the primary cause of the hemorrhagic event with an underlying DVA. Magnetic resonance imaging is usually the diagnostic tool of choice to evaluate DVAs. In cases of isolated cavernoma, conservative treatment with periodic clinical monitoring and imaging is sufficient. This differs from cases that present with hemorrhage, in which case surgical intervention might be considered depending on the extension. It is important for physicians to be aware of the association of cavernous malformations and developmental venous anomalies.
